# Blood flow reversal from the external into the internal carotid artery—New insights into the hemodynamics at the carotid bifurcation

**DOI:** 10.1002/brb3.1139

**Published:** 2018-10-12

**Authors:** Patrick Meyer, Johann Otto Pelz

**Affiliations:** ^1^ Department of Neurology University Hospital Leipzig Leipzig Germany

**Keywords:** blood flow reversal, carotid bifurcation, ischemic stroke, vector flow imaging

## Abstract

**Background:**

Complex blood flow patterns are a well‐known phenomenon at the carotid bifurcation. However, unlike for the descending aorta, a blood flow reversal has not been detected at the carotid bifurcation, so far.

**Methods:**

In 17 subjects, flow patterns with focus on blood flow reversal were examined at the carotid bifurcation with vector flow imaging.

**Results:**

We found a blood flow reversal from the external carotid artery (ECA) into the internal carotid artery (ICA) in 13 of 25 (52%) carotid bifurcations. The blood flow reversal ranged 5.3 ± 1.7 mm (range 2.6–8.3 mm) distally to the beginning of the ECA and lasted 105 ± 59 ms (range 32–225 ms). The mean peak systolic velocity within the blood flow reversal was 12.5 ± 4.6 cm/s (range 5–18 cm/s).

**Conclusion:**

A blood flow reversal from the ECA into the ICA during the systole is a frequent finding at the carotid bifurcation. Considering ischemic stroke, retrograde embolism from plaques in the proximal ECA into the ICA might play a role.

## 1 INTRODUCTION

Applying the TOAST criteria (Trial of ORG 10172 in Acute Stroke Treatment), 23% of ischemic strokes are caused by a large artery atherosclerosis, that is an at least 50% stenosis of the predominantly extracranial internal carotid artery (ICA; Ornello et al., 2018). Therefore, current guidelines state that for patients with ischemic stroke in the carotid territory noninvasive imaging of the cervical vessels should be performed routinely (Powers et al., 2018). So far, this noninvasive imaging by computer tomography angiography, magnetic resonance angiography or color‐coded duplexsonography (CDS) has exclusively focused on the common carotid artery (CCA) and the ICA. There are only case reports describing an embolism from the external carotid artery (ECA) as the cause of ischemic stroke in patients with no coexisting ipsilateral ICA stenosis (Nicolas, Hubert, Leclère, Etienne, & Robert, 2016; Weinberger, Robbins, & Jacobson, 1983). Recently, Harloff and colleagues described a blood flow reversal in the aorta which might lead to a retrograde embolism from the descending aorta into the outlet of the brain supplying arteries (Harloff et al., 2009). Aim of this study was to examine whether such a blood flow reversal might also appear at the carotid bifurcation, thus, linking the ECA with the vascular territory of the ICA.

## 2 METHODS

The study was approved by the local ethics committee (reference no.: 503/16‐ek). All participants provided informed and written consent prior to the commencement of the study.

**Figure 1:**
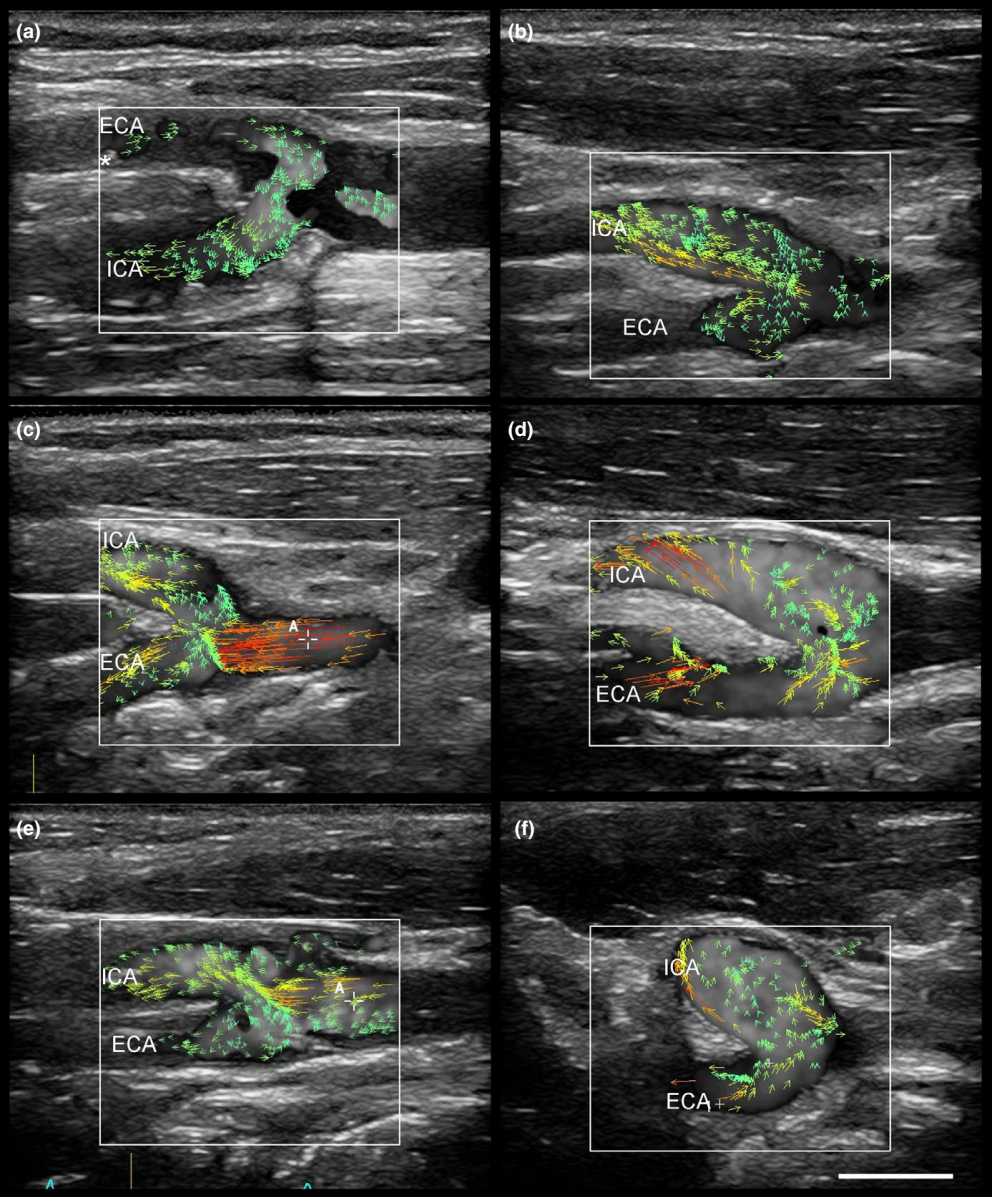
Blood flow reversal from the external (ECA) into the internal carotid artery (ICA, a‐f). As demonstrated in (a), the blood flow reversal connects a plaque (marked by asterisk) in the proximal ECA with the origin of the ICA. The vector arrows indicate the direction of blood flow. Scale bar: 1 cm

Subjects were eligible if, at least at one side, the three carotid arteries constituting the carotid bifurcation could be visualized at a single sagittal plane (Figure 1).

Seventeen subjects (12 female, mean age 52 ± 19 years) participated in this study. Nine participants were recruited from our neurovascular ultrasound outpatient clinic where they were followed up because of a prior ischemic stroke, three patients were recruited from our stroke unit and five subjects were healthy volunteers without a history of any vascular disease.

### 2.1 Vector flow imaging

Vector flow imaging (VFI) is an angle‐independent new ultrasound technology based on pulsed wave imaging. After a series of single unfocused sonographic beams are transmitted, multiple image receiving lines are obtained. Although every single image generated is affected by low contrast, a significant quality improvement can be achieved by coherently compounding multiple plane wave images. VFI allows the calculation of the true velocity vectors at any location into a vessel and shows velocity vectors, streamline distribution, and vorticity distribution at high frame rates. (Ekroll, Dahl, Torp, & Løvstakken, 2014; Goddi, Bortolotto, et al., 2017; Goddi, Fanizza, et al., 2017).

Vector flow imaging was performed with a Mindray Resona 7 (Shenzhen Mindray Biomedical Electronic Co, Shenzhen, China) equipped with a linear transducer (L9‐3U). The blood flow was analyzed by the system for 1.5 s at a pulse repetition frequency of 13 kHz and a frame rate of 533 Hz, which allowed to study at least one complete cardiac cycle. Practically, all participants were lying in a supine position with the sonographer sitting behind them. The examination started in the B‐mode with the depth set to 4 cm. The carotid bifurcation with the distal CCA, the proximal ECA, and the proximal ICA was visualized in one sagittal plane. Subsequently, the ultrasound mode was switched to the VFI mode, and the subjects were asked not to breathe or swallow during the scan which lasted 1.5 s. Each carotid bifurcation was examined three times, and every scanning was saved as a video file.

### 2.2 Analyses of the vector flow imaging recordings

Measurements were conducted *online* at the ultrasound system. At first, each VFI recording was analyzed for the presence of a blood flow reversal from the ECA into the ICA, and if present, its occurrence during the heart cycle was determined. Subsequently, the blood flow reversal was further quantified in terms of its duration, maximum distance from the beginning of the ECA, and mean peak systolic flow velocity. The median of the three measurements was used for statistical analyses which were performed using SPSS (version 24.0, IBM Corporation, New York, NY).

## 3 RESULTS

In 25 of 34 carotid bifurcations, all three vessels could be visualized at one sagittal plane. We detected a blood flow reversal from the ECA into the ICA in 13 of 25 (52%) carotid bifurcations. Twelve of 13 (92%) flow reversals from the ECA into the ICA occurred during the incisure at the end of the systole and exhibited a vortex flow pattern (Figure 1). In one case, the flow reversal was detected during the end of the diastole. The blood flow reversal was located at the outer wall of the ECA, ranged 5.3 ± 1.7 mm (range 2.6–8.3 mm) distally to the beginning of the ECA and lasted 105 ± 59 ms (range 32–225 ms). The mean peak systolic velocity within the flow reversal was 12.5 ± 4.6 cm/s (range 5–18 cm/s).

## 4 DISCUSSION

Examining the carotid bifurcation with VFI, we found a blood flow reversal from the proximal part of the ECA into the ICA during the systole in 52% of cases. Generally, flow disturbances at the carotid bifurcation are a common and physiological phenomenon, mainly caused by the complex anatomy of the branching vessels with changes in their diameters and their vessel wall architecture (Anayiotos & Papaharilaou, 2012).

So far, visualization and profound analysis of flow patterns at the carotid bifurcation has been limited by the low temporal resolution of CDS with about 15–20 images per second. The temporal resolution of VFI with 500–600 images per second exceeds the temporal resolution of CDS approximately by the factor 30. As shown by Goddi and colleagues, VFI could better visualize the complex blood flow at the carotid bifurcation and discriminate multiple complex flow patterns like flow separation, recirculation, counter‐eddies, multiple eddies, and vortexes (Goddi, Bortolotto, et al., 2017; Goddi, Fanizza, et al., 2017; Goddi et al., 2018). The blood flow reversal from the ECA into the ICA now extends this spectrum of complex flow patterns at the carotid bifurcation. It seems to be physiological since it was detected in young subjects without a history of vascular disease as well as in patients who had suffered an ischemic stroke. Our results also confirmed the findings of Ekroll and co‐workers who were the first to visualize and describe such a blood flow reversal from the ECA into the ICA in a single healthy proband (Ekroll et al., 2014). While they did not examine this flow reversal systematically, we could demonstrate that its duration would be long enough to allow an embolus from a plaque within the proximal 8 mm of the ECA to reach the outlet of the ICA (Figure 1a).

Therefore, our finding now also extends the knowledge about potential sources and routes of arterial embolization into the brain. Noteworthy, such a blood flow reversal was also demonstrated for the descending aorta. Harloff and colleagues measured a mean retrograde diastolic flow of 26 mm in the descending aorta which was sufficient to connect complex plaques of the descending aorta with the outlet of the left subclavian artery, the left CCA, or the brachiocephalic trunk (Harloff et al., 2010, 2009).

Thus, further studies are warranted that focus on the relationship between ECA plaques and ischemic stroke. However, this might be challenging because complex plaques in the descending aorta—and vice versa probably also in the ECA—are presumably a marker of generalized atherosclerosis and high vascular risk (Katsanos et al, 2014). From the clinical experience of the authors, ischemic stroke patients with isolated plaques in the ECA and an otherwise cryptogenic stroke are very rare. This is mainly because the pathophysiological mechanisms that cause and facilitate the formation of plaques are evident at the entire carotid bifurcation Anayiotos & Papaharilaou, 2012). Therefore, the establishment of a true causal relationship of ECA plaques with ischemic stroke through retrograde embolism would require a thorough approach and might suffer from many inherent limitations. Moreover, even if such a causal relationship could be established at an individual level, this would probably not change the secondary prevention with inhibitors of platelet aggregation and statins as the medication of choice.

This study has some limitations. At first, our findings need to be confirmed in a larger cohort of ischemic stroke patients. Secondly, it should be assessed whether the duration and length of the blood flow reversal could be influenced, for example, by blood pressure, heart rate, or physical exercise. A decreasing heart rate was shown to correlate with an increased retrograde flow length in the proximal descending aorta (Harloff et al., 2010). Furthermore, since the blood flow reversal from the ECA into the ICA mainly occurred during the incisure at the systole which is caused by the short retrograde flow in the proximal aorta due to the closure of the aortic valve, it might be more pronounced in patients with aortic valve insufficiency. Thirdly, the normal blood flow at the carotid bifurcation has already a complex 3D shape. Since VFI only assesses the blood flow in 2D, complex flow patterns might be missed, and therefore, the frequency of blood flow reversal might even be higher.

## 5 CONCLUSIONS

A blood flow reversal from the ECA into the ICA during the systole is a frequent finding at the carotid bifurcation. Considering ischemic stroke, retrograde embolism from plaques in the proximal ECA into the ICA might play a role.

## CONFLICT OF INTEREST

None declared.
